# Metabolic Risk Factors, Leisure Time Physical Activity, and Nutrition in German Children and Adolescents

**DOI:** 10.1155/2012/370850

**Published:** 2012-06-19

**Authors:** Gerda-Maria Haas, Evelyn Liepold, Peter Schwandt

**Affiliations:** ^1^Atherosclerosis Prevention Institute, Munich-Nuremberg, 81477 Munich, Germany; ^2^Ludwig-Maximilians-University Munich, 80539 Munich, Germany

## Abstract

*Purpose*. We assessed the five components of the metabolic syndrome (MetS) as defined by the International Diabetes Federation (IDF) in 6040 (3158 males) youths aged 6–16 years who participated in the Präventions-Erziehungs-Programm (PEP Family Heart Study) in Nuernberg between 2000 and 2007. The purpose of this cross-sectional study was to examine associations with lifestyle habits. *Results and Discussion*. The prevalence of MetS was low in children (1.6%) and adolescents (2.3%). High waist circumference (WC) and low HDL-C were slightly higher in females (9.5% and 7.5%, resp.) than in males (8.8% and 5.7%, resp.). Low leisure time physical activity (LTPA) was significantly associated with low HDL-C (odds ratio [OR] 2.4; 95% CI 1.2–5.0) and inversely associated with hypertension (*r* = −0.146), hypertriglyceridemia (*r* = −0.141), and central adiposity (*r* = −0.258). The risk for low HDL-C (≤1.3 mmol/L) was 1.7-fold (CI 1.0–2.6) higher in youth with high (≥33%) saturated fat consumption. A low polyunsaturated/saturated fat ratio (P/S ratio) was significantly associated with fasting hyperglycemia (OR 1.4; 95% CI 1.0–1.2).

## 1. Introduction

 The metabolic syndrome (MetS) in youths is no more limited to industrialized countries, and ethnic disparities are well documented [1, 2]. Because MetS represents a serious risk of cardiovascular disease, tracking into young adulthood early intervention using healthy lifestyle is mandatory [3, 4]. Dietary intake has been linked to individual components of MetS suggesting that a western dietary pattern promotes the incidence of MetS [5]. Increase in moderate to vigorous physical activity was associated with better cardiometabolic risk factors in youths [6]. The purpose of this cross-sectional study was to examine associations between MetS components and lifestyle factors such as nutrition and physical activity in a large sample of healthy children and adolescents. 

## 2. Material and Methods

 We investigated 2393 children (1220 males) aged 6 to <10 years and 3647 adolescents (1938 males) aged 10 to <16 years participating in the community-based Praeventions-Erziehungs-Programm (PEP) Family Heart Study between 2000 and 2007. Regularly trained research assistants measured height, weight, waist circumference (WC), systolic blood pressure (SBP), diastolic blood pressure (DBP), fasting triglycerides (TG), high-density cholesterol (HDL-C), and fasting plasma glucose (FPG) using standardized methods as described previously [7–9].

 Parents and their children were trained to precisely document their daily intake of food and beverage on seven consecutive days including a weekend [9]. Each participating family was issued an accurately calibrated digital food scale (Soehnle Combi Plus, Nassau, Germany) to document their weighted protocols day by day. Completed dietary records were analyzed by trained dieticians using the computer program PRODI (version 4.5, Nutri-Science, Freiburg, Germany), which includes “Deutscher Lebensmittelschlüssel” supplemented with individual special items. The American Standard Code transferred the PRODI data for Information Interchange (ASCII) into SPSS (version 15.0) for documentation and calculation.

 Self-reported physical activity (PA) and sedentary behavior (SB) were assessed by assisted questionnaires as previously described [9]. SB was defined as typical behavior requiring low levels of energy expenditure to perform (1.0–1.5 METs). The participants were asked to register how often during the last seven days they performed leisure time sport activities (LTPAs) out of 39 listed items and sedentary activities (including computer use, playing video games, viewing television, videotapes, listening to music, reading, quiet sitting, sleeping, lying down, etc.) for at least 15 minutes. PA was calculated as the product of the duration and frequency of each activity (in hours per week). The time per week spent on each activity was multiplied by its typical energy expenditure in terms of metabolic equivalents of task (MET) and then summed over all activities to yield MET hours per week. We used special compendia for children and adolescents. LTPA was categorized into light, moderate (heart rate above rest and breathing somewhat harder than normal), and vigorous (heart rate considerably above rest and breathing much harder than normal) activities and MET levels presented for each effort level. As examples, leisurely, moderate-effort bicycling was assigned 6.2 METs, and moderate walking was considered as 3.6 METs [6, 10].

We used the IDF cut-offs for the five MetS components in terms of WC ≥ 90th percentile, SBP ≥ 130 or DBP ≥ 85 mm Hg, TG ≥ 1.7 mmol/L, HDL-C ≤ 1.03 mmol/L, and glucose ≥ 5.6 mmol/L [11].

 For statistical analyses, we used SPSS 18.0 considering  *P* < 0.05 significant. Descriptive results are expressed as a mean for continuous variables and percentages for categorical variables. Differences between genders were tested by analysis of variance. Multiple linear regression models were calculated using all variables for LTPA. Multivariate logistic regression models were calculated using macronutrients as percentage of energy fat and consumption, respectively. 

## 3. Results and Discussion

**Figure 1:**
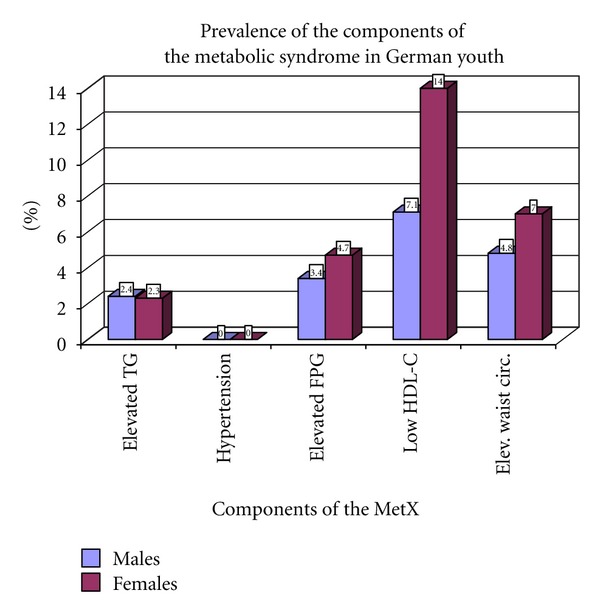


 Mean values of WC, BP, HDL-C, and FPG were significantly higher in males than in females, whereas TG was significantly higher in females of both age groups (Table 1). As shown in Figure 1, the prevalence of the metabolic syndrome consisting of all five components was very low in children (1.4% in boys and 1.7% in girls) and adolescents (2.8% in males and 1.7% in females).

 LTPA was higher in boys (26.3 METs) than in girls (15.5 METs) and higher in male (35.8 METs) than in female (21.2 METs) adolescents. We observed a significant (*P* < 0.001) association between low HDL-C and sports less than 30 minute/day (OR 2.4; 95% CI 1.2–5.0). Low LTPA was significantly and inversely associated with elevated SBP (*r* = −0.446) and elevated TG (*r* = −1.087). We found the strongest associations of high WC with low sport activity (*r* = −0.749) and with sedentary time (*r* = 0.307), and sedentary time was significantly and inversely associated with high HDL-C (*r* = −0.903). This is consistent with a recent meta-analysis of 14 studies with 20.871 youths showing significant and inverse associations between moderate to vigorous physical activity and HDL-C [6].

 Mean absolute daily intake of energy (Kcal) and of fat was higher in male than in female adolescents but was similar in terms of percentage of energy. Energy intake was inversely associated with HDL-C (*r* = −0.243). The risk of low HDL-C (≤1.3 mmol/L) was 1.7-fold (CI 1.0–2.6) higher in children and adolescents consuming much (≥33%) saturated fat compared with low SAFA intake. Significant associations have been observed between FPG >5.6 mmol/L and a low P/S ratio <0.35 (OR 1.4; CI 95% 1.0–2.1). The probability of high FPG was 10.3 (CI 2.9–36.2) times higher in youths consuming more than 35% fat than in youths consuming less fat. A comparison with the ARIC Study [4] is difficult because of different assessment of dietary intake (7-day weighed food versus dietary patterns).

 A limitation is the cross-sectional design of the study, which should be confirmed by longitudinal data, which is not realistic for this setting. Vice versa, the strength of this study is to organize for more than 6.000 children and adolescents submitting weighed dietary protocols over 7 days and documenting simultaneously their physical leisure time activity as performed by the families participating in the PEP Family Heart Study. 

## 4. Conclusions

 The prevalence of components of the metabolic syndrome as defined by the International Diabetes Federation definition is low in this sample of 6040 healthy children and adolescents. We found significant associations between MetS components and leisure time physical activity including sedentary behaviour and nutrition. This suggests that sustained lifestyle change in families participating in the PEP Family Heart Study might reduce even low cardiometabolic risk in their children. However, longitudinal studies should confirm this preventive approach.

## Figures and Tables

**Table 1:** Characteristics of 2393 children and of 3647 adolescents;  *P < 0.05 significant between genders.

(*n*)	Boys (1220)		Girls (1173)		Males (1938)		Females (1709)	
	mean	SD	mean	SD	mean	SD	mean	SD
Age (years)	8.7	1.7	8.7	1.7	14.3	1.9	14.3	1.9
Waist circumference (cm)	62.0*	7.5	60.7	7.5	74.0*	9.3	71.4	9.3
SBP (mm Hg)	104.7*	8.5	103.7	8.6	114.7*	11.5	109.5	9.3
DBP (mm Hg)	67.0*	7.6	66.1	7.7	71.5*	8.0	69.7	7.8
Total cholesterol (mmol/L)	4.7	0.8	4.8*	0.8	4.4	0.8	4.6*	0.8
LDL-C (mmol/L)	2.7	0.7	2.9*	0.7	2.5	0.7	2.6*	0.7
HDL-C (mmol/L)	1.7*	0.3	1.6	0.3	1.5	0.3	1.6*	0.3
Triglycerides (mmol/L)	0.7	0.3	0.8*	0.3	0.8	0.3	0.9*	0.4
Fasting plasma glucose (mmol/L)	5.2*	0.6	5.1	0.7	5.3*	0.6	5.1	0.6
